# Supplementary material for: A universal packaging substrate for mechanically stable assembly of stretchable electronics
Source: Nat Commun. 2024 Jul 19;15:6106. doi: 10.1038/s41467-024-50494-8 (PMC11271615; doi:10.1038/s41467-024-50494-8)
Supplement: Supplementary file 1 — Supporting Information [file 41467_2024_50494_MOESM1_ESM.pdf]

A Universal Packaging Substrate for Mechanically Stable Assembly of Stretchable Electronics

Yan Shao[1,2#], Jianfeng Yan[1#], Yinglin Zhi[1], Chun Li[1], Qingxian Li[3], Kaimin Wang[1], Rui Xia[1], Xinyue Xiang[1], Liqian Liu[1], Guoli Chen[1], Hanxue Zhang[1], Daohang Cai[1], Haochuan Wang[1], Xing Cheng[1], Canhui Yang[3], Fuzeng Ren[1*], Yanhao Yu[1,4*]

[1]Department of Materials Science and Engineering, Southern University of Science and Technology, Shenzhen 518055, China

[2]School of Materials Science and Engineering, Yancheng Institute of Technology, Yancheng 224051, China

[3]Shenzhen Key Laboratory of Soft Mechanics & Smart Manufacturing, Department of Mechanics and Aerospace Engineering, Southern University of Science and Technology, Shenzhen 518055, China

[4]Guangdong Provincial Key Laboratory of Sustainable Biomimetic Materials and Green Energy, Southern University of Science and Technology, Shenzhen 518055, China.

[*] E-mail: yuyh@sustech.edu.cn; renfz@sustech.edu.cn

[#] Contributed equally to this work

This file includes:

Supplementary methods

Supplementary Figures: Fig. S1 to S22

Supplementary Tables: Table S1 to S3

Supplementary References

Other Supplementary Materials for this manuscript include the following:

Supplementary Movies S1 to S5

Supplementary Methods

Materials

Polypropylene grafted by 8-10 wt% maleic anhydride (PP-g-MAH) with an average molecular weight (Mw) of 9100, and toluene were purchased from Sigma-Aldrich. Poly (styrene-block-isobutylene-block-Styrene) (SIBS, SIBSTAR 102T) with 15 wt% of polystyrene and poly (styrene-block-isobutylene-block-Styrene) (SIBS, SIBSTAR 103T) with 30 wt% of polystyrene was purchased from Kaneka Corporation. Poly(dimethylsiloxane) (PDMS, Sylgard 184) was purchased from Dow Corning. 2-aminobenzothiazole, (3-aminopropyl) triethoxysilane (APTES) and chitosan power were purchased from Aladdin Co., Ltd. Poly(3,4-ethylenedioxythiophene): poly(styrenesulfonate) (PEDOT:PSS, Clevios PH1000) was purchased from Heraeus Co. Polyethylene terephthalate (PET) sheets were purchased from Teijin corporation in Japan. Polyisobutylene (PIB) oligomer with average molecular weight (Mw) of 1300 was supplied by Macklin.

The optimization of the soft/hard interface

The optimization of the soft/hard interface shown in Fig. S7 was done by controlling the toluene evaporating time of the hard region. In specific, 4.5 mL of 250 mg/mL SIBS (30 wt% PS)/toluene solution was dropped in the left part of the rectangular container. A PDMS film was inserted in the right part to leave the space for the soft component. The solidification degree of the hard region was manipulated by varying the toluene evaporation at room temperature in the fume hood. After the toluene evaporated for different times ranging from 0 to 24 h, the PDMS spacer was removed and the blank space was filled with 4 mL SIBS/PIB/toluene solution. The hard-soft (HS) films with different transition interfaces were obtained after the evaporation of toluene.

Fabrication of the stretchable circuit

The stretchable conducting lines were fabricated using a composite of SIBS, PIB, and liquid metal. Initially, 1.35 g of liquid metal was introduced into 30 mL of acetone. This mixture underwent ultrasonication at 44% amplitude for 20 mins in a water bath at room temperature to generate liquid metal microdroplets. Subsequently, the microdroplets were isolated via centrifugation at 519 × g for 20 mins, and the solvent was carefully decanted. The resulting liquid metal microdroplets were then combined with 1 mL of SIBS/PIB/toluene solution (100 mg/mL) before being deposited onto a glass plate as ink. Upon complete evaporation of the toluene solvent, a composite film comprising SIBS, PIB, and liquid metal was obtained. This film was cut into strips and affixed to the soft region of the prepared SIBS substrate to serve as conducting lines. To

increase their conductivity, an acoustic field was applied to the conducting lines using a probe sonicator at 30% amplitude. The stiff modules, including LED, inductor, IC chip, diode, and resistors, underwent treatment with oxygen plasma at 300 W for 5 mins. Subsequently, their surfaces were treated with a solution of ethanol and water (9:1 volume ratio) containing 5 wt% APTE, followed by drying at 80 °C for 1 hour. The treated modules were integrated onto module-specific SIBS substrates with an interposer layer.

Characterizations

Confocal laser scanning microscopy: Confocal fluorescence images were acquired on a Zeiss LSM 980 instrument. The top-view confocal images were obtained by a raster scan at 400 Hz on the x-y plane with a 5×air objective. The images were collected at a resolution of 1024 × 1024 pixels and a depth of 16-bit. The SIBS/PIB/toluene solution was dyed with coumarin 6. The horizontal Z-stacked images of soft-hard were acquired by the reconstruction of cross-sectional images. All of the confocal images were analyzed using Zen Blue 3.0 software.

Differential scanning calorimetry (DSC): DSC measurements were performed on DSC1 (Mettler Toledo) with a lowest temperature of -60 °C. The samples were heated to 150 °C to remove heat history and quenched at 50 °C for 30 mins. About 10 mg of the sample was used for the measurement. The sample was maintained at -60 °C for 10 mins, and then heated from -60 °C to 120 °C with a heating rate of 10 °C/min under continuous nitrogen purge.

Weight retention measurement of SIBS packaged hydrogel*:* The water retention was evaluated by exposing the packaged hydrogels at room temperature. The weight of the hydrogels was recorded at regular intervals of 7 days by an electronic scale. The weight retention ratio at time t (WR_t) was calculated by the following equation:

$$WR_t = \frac{W_t}{W_0} \times 100\%$$

where W_t and W_0 represent the weight of the hydrogel at time t and 0, respectively. At least three samples were tested. The data was presented as mean values with mean absolute deviations.

Supplementary Figures

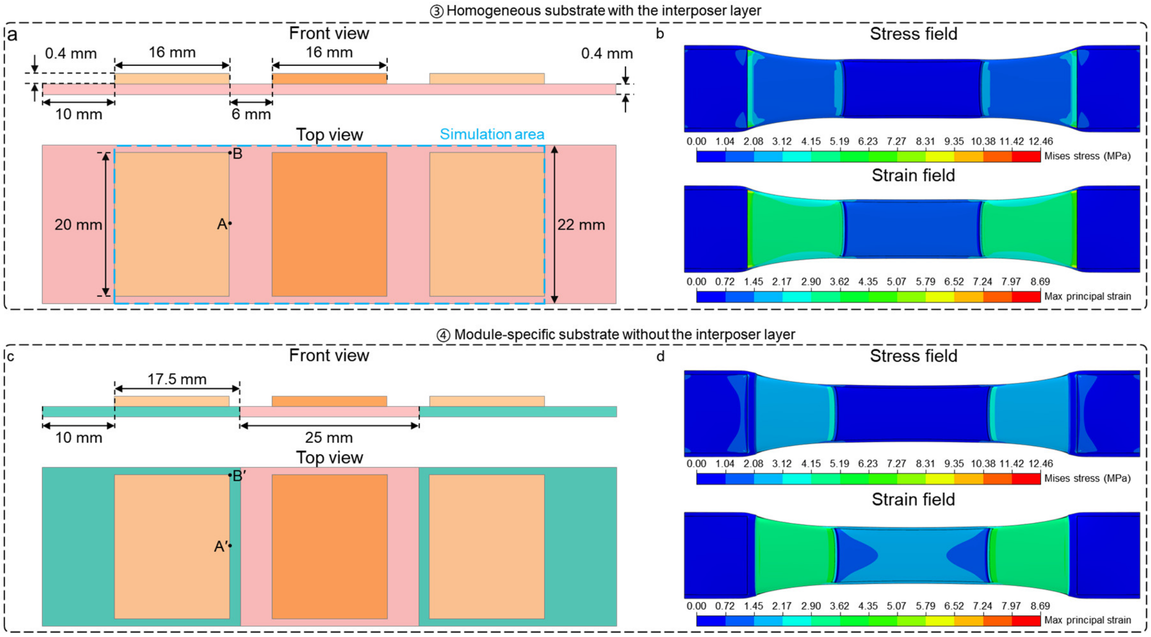

Fig. S1. Finite element simulation. **(a)** Schematics, and **(b)** finite element simulation results of the stretchable device based on an all-soft substrate. The Mises stresses and the max principal strains of the substrate at point A and point B are σ_A = 3.99 MPa, σ_B = 12.46 MPa, ε_A = 4.42, ε_B = 8.69, respectively. **(c)** Schematics, and **(d)** finite element simulation results of the stretchable device based on a heterogeneous substrate. The Mises stresses and the max principal strains of point A′ and point B′ are $\sigma_{A'}$ = 1.04 MPa, $\sigma_{B'}$ = 4.65 MPa, $\varepsilon_{A'}$ = 0.32, $\varepsilon_{B'}$ = 1.28, respectively. The nominal strain of the middle segment (25 mm in length) is 200% in the simulation. The scale ranges for the stress and strain fields were unified to 12.46 MPa and 8.69 for a clear comparison, respectively.

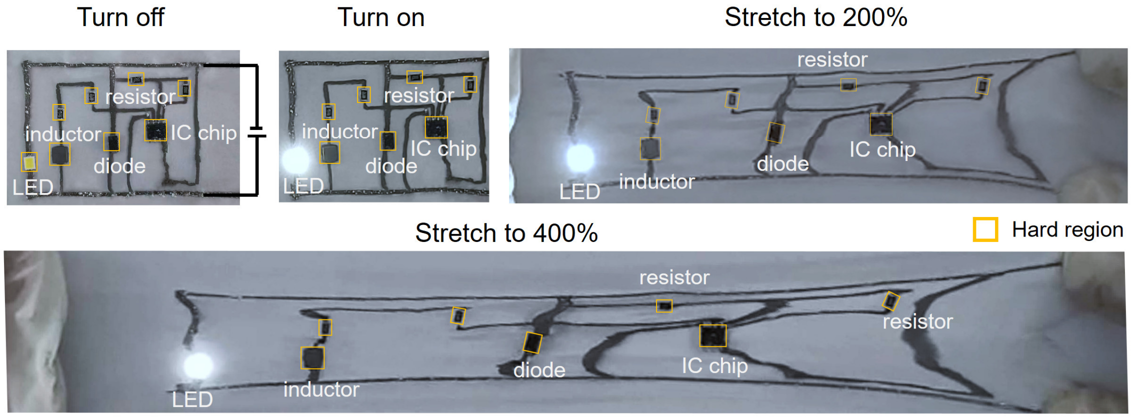

Fig. S2. Digital images showing the mechanical and electrical stabilities of an actual operating circuit under large stretch.

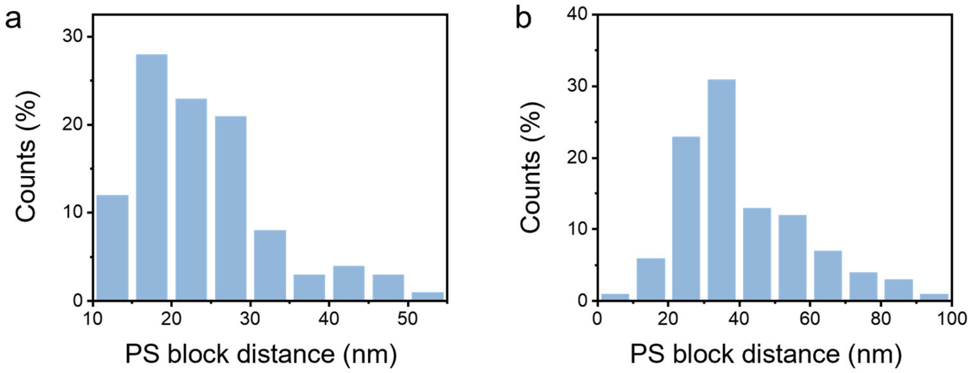

Fig. S3. Statistical results of PS block distance for **(a)** pristine SIBS and **(b)** SIBS/PIB blend. The weight percentage of PS is 15%.

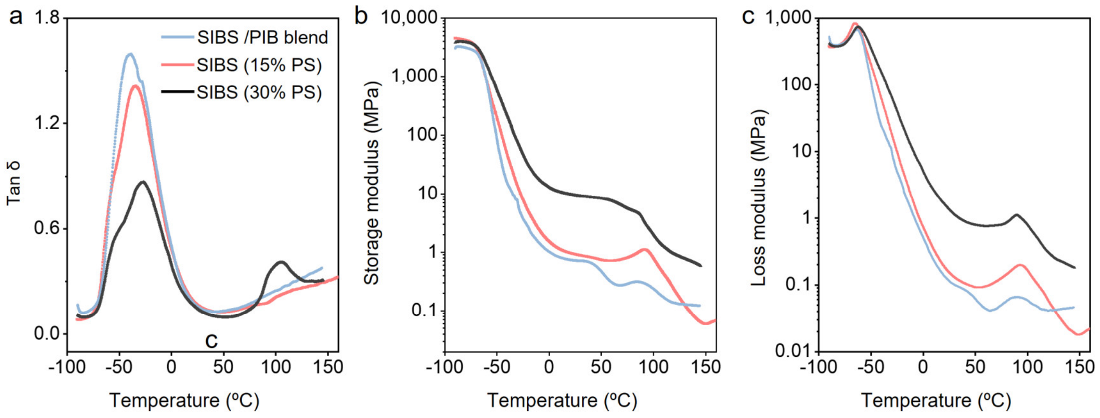

Fig. S4. Dynamic mechanical analysis (DMA) for pristine SIBS with 30 wt% of PS, pristine SIBS with 15 wt% of PS, and SIBS/PIB blend. **(a)** Tan δ spectra, **(b)** storage modulus, **(c)** loss modulus as a function of temperature. SIBS represents poly(styrene-isobutylene-styrene); PIB represents polyisobutylene.

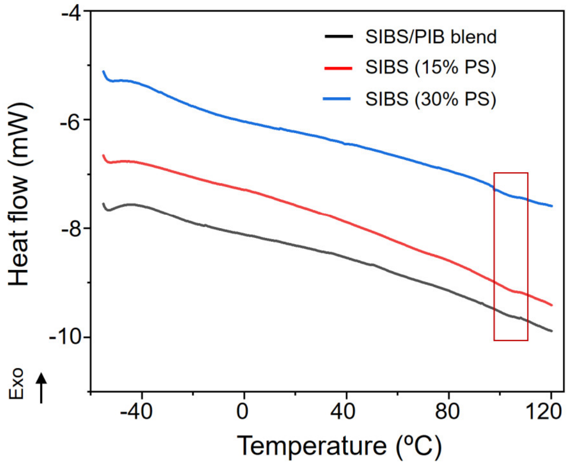

Fig. S5. DSC curves of pristine SIBS with 30 wt% of PS, pristine SIBS with 15 wt% of PS, and SIBS/PIB blend. The rectangular highlights the T_g peaks of PS.

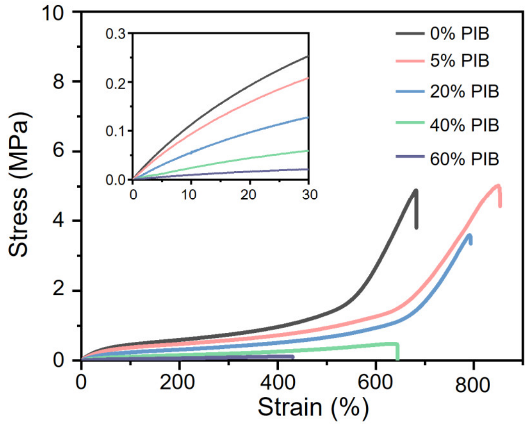

Fig. S6. Stress-strain curve of the SIBS/PIB blend film, showing the decrease of modulus as the increase of PIB content. The inset is a magnified illustration for the region within 30% strain. The elastic modulus was determined from the slope within the linear range shown in the inset.

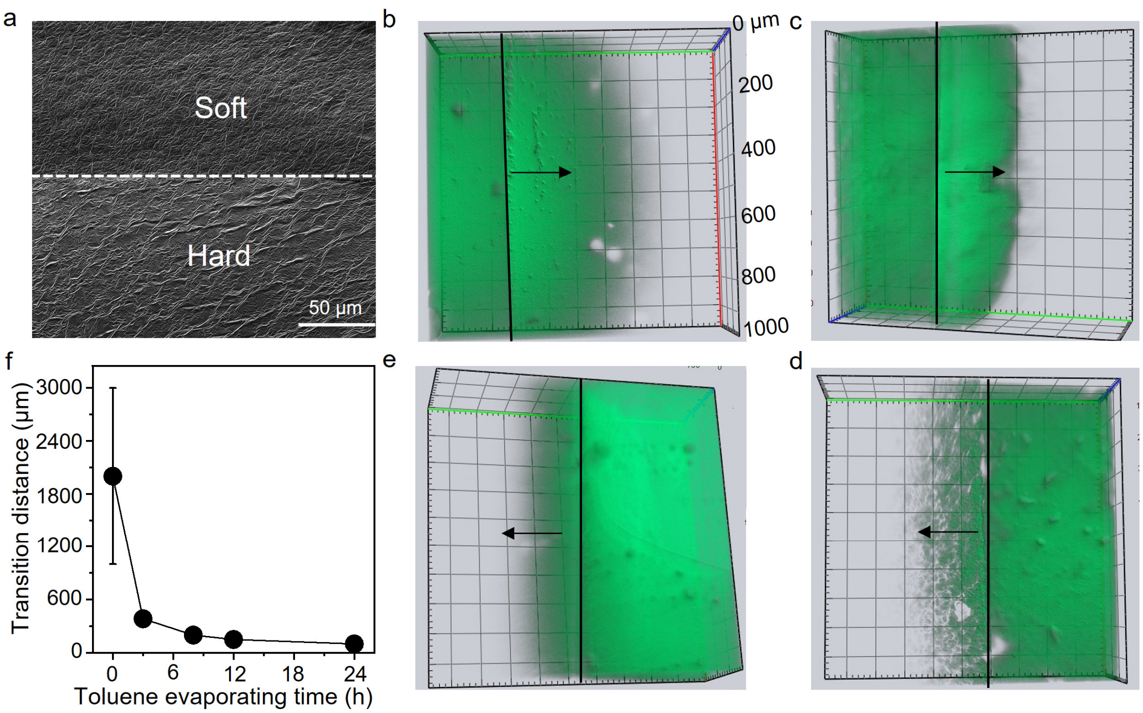

Fig. S7. Optimization of the interface between the soft and hard regions. **(a)** Top-view SEM image of the interface, soft refers to the soft region; hard refers to the hard region. Representative results are presented from $n = 5$ independently repeated experiments. **(b-e)** 3D confocal fluorescence images of the interfaces obtained at different evaporation times for the hard region: (b) 3 h, (c) 8 h, (d)12 h, and (e) 24 h. The soft region was dyed in green and the hard region was transparent. **(f)** Transition distance between the soft and hard regions at a function of toluene evaporating time measured from the confocal fluorescence images in (b-e). The error bar represents standard deviations and data is from $n = 3$ biologically independent samples and expressed as mean ± s.d.

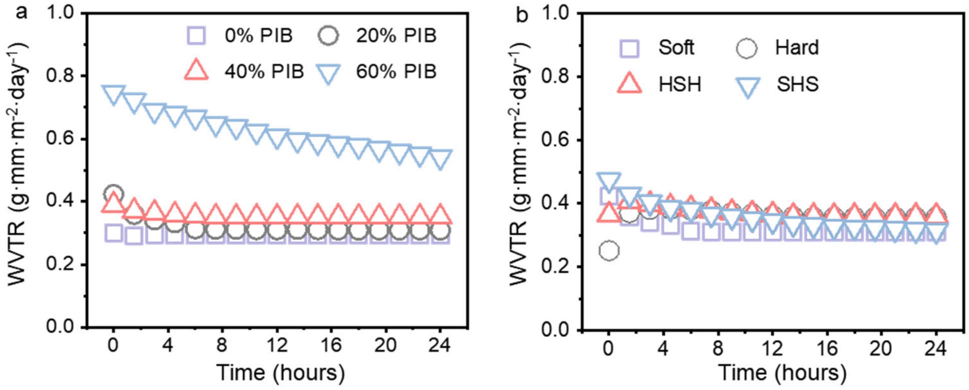

Fig. S8. Raw data of WVTR versus testing time. **(a)** WVTR of the SIBS/PIB blend films with different PIB content. **(b)** WVTR of the soft, hard, HSH and SHS films. The soft portion was the SIBS/PIB blend with 20% of PIB. All data were collected at 38 °C and 90% RH. Hard and soft

refer to the hard SIBS and the soft SIBS, respectively; HSH and SHS refer to the hard-soft-hard SIBS and the soft-hard-soft SIBS, respectively.

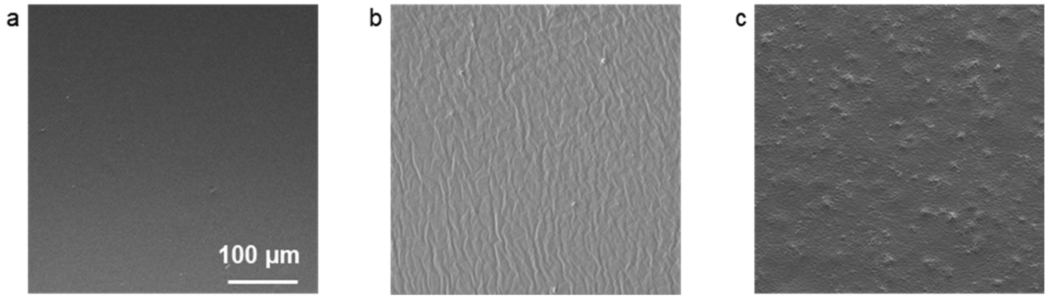

Fig. S9. Top-view SEM images of **(a)** the pristine PDMS (polydimethylsiloxane) film, **(b)** the PDMS film treated by O_2 plasma and APTES ((3-aminopropyl) triethoxysilane), and **(c)** the PDMS film coated with the SIBS/PP-g-MAH (maleic anhydride grafted polypropylene) interposer layer. Representative results are presented from $n = 5$ independently repeated experiments.

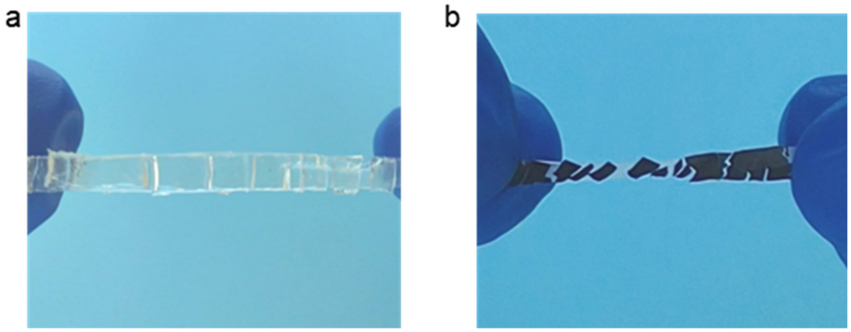

Fig. S10. Photographs of **(a)** hydrogel and **(b)** PEDOT:PSS adhered on a SIBS film being stretched, showing the interfacial toughness of these surfaces exceeded the cohesive energy of hydrogel and PEDOT:PSS ((poly(3,4-ethylenedioxythiophene):poly(styrene sulfonate)).

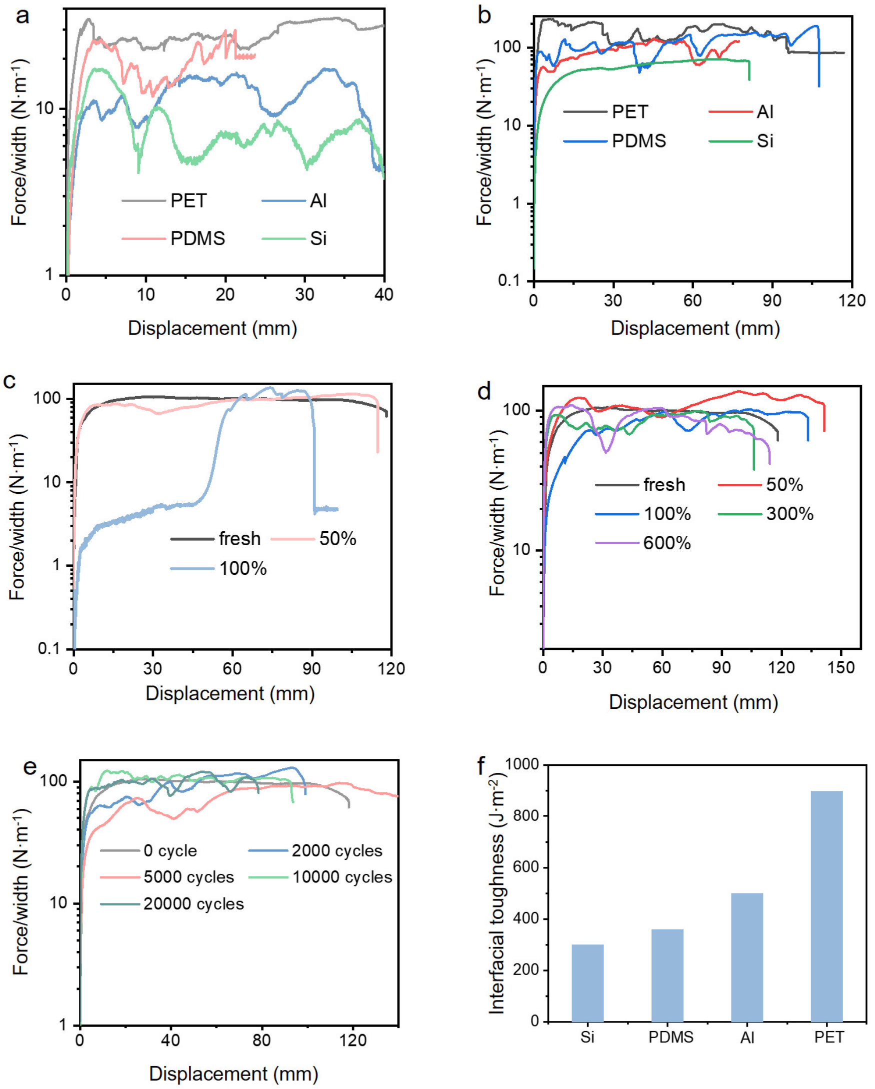

Fig. S11. Adhesion property of the packaging substrate. **(a,b)** Force/width–displacement curves of the APTES-treated Al (aluminum), Si (silicon), PDMS (polydimethylsiloxane), and PET (polyethylene terephthalate) adhered on the packaging substrate without **(a)** and with **(b)** the interposer layer. **(c)** Force/width–displacement curves of the APTES-treated Al adhered on the packaging substrate without the interposer layer after being stretched to 0%, 50% and 100% strain by linear motor. **(d)** Force/width–displacement curves of the APTES-treated Al adhered on the packaging substrate with the interposer layer after being stretched to 0%, 50%,100%, 300% and 600% strain by linear motor. **(e)** Force/width–displacement curves of APTES treated Al pressed on the packaging substrate after being stretched to 100% strain by linear motor for 0, 2000, 5000, 10000 and 20000 cycles by the linear motor. Adhesions in **(a-e)** were pressed by 200 g weight at 80 °C. **(f)** Interfacial toughness of the APTES-treated PDMS, Al, Si and PET adhered the packaging substrate by hot-pressing at 150 °C.

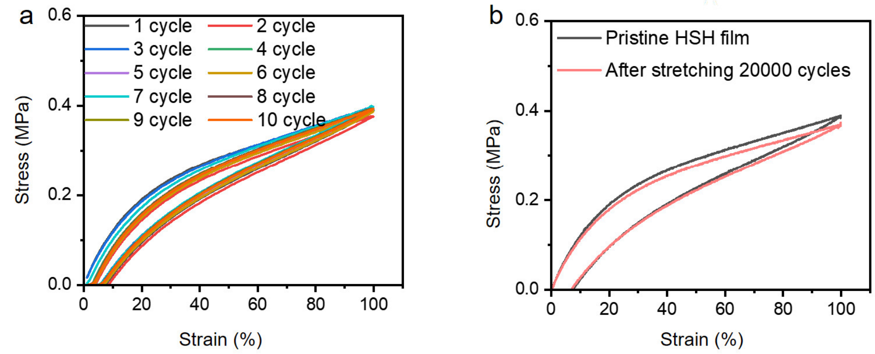

Fig. S12. (a) Tensile stress-strain curves of the HSH film being cyclically stretched to 100%. **(b)** Tensile strain-stress curves of the HSH film before and after being stretched for 20, 000 times to 100% by a linear motor.

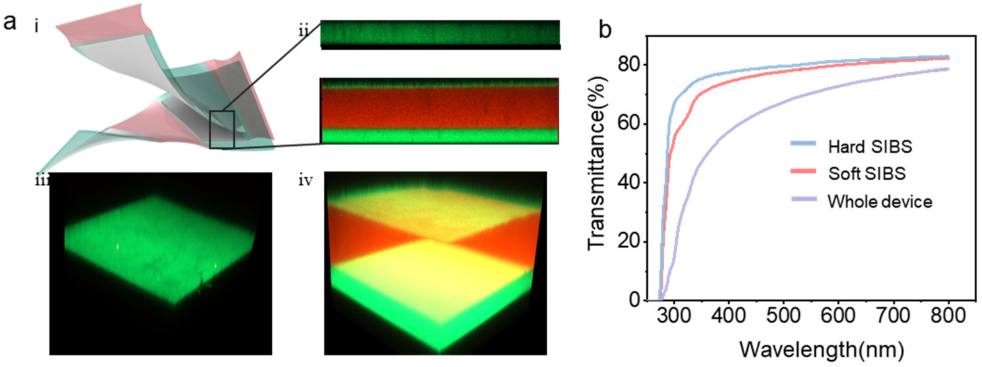

Fig. S13. (a) Schematic **(i)** and fluorescence images **(ii-iv)** of the arbitrarily deformable bioelectronic device. **(ii)** There were 5 layers in total: hard region of the SHS packaging substrate, PDMS tribo-layer (non-fluorescent), SIBS tribo-layer, conductive hydrogel layer, and the hard region of HSH packaging substrate. **(iii)** 3D images of the hard region of the HSH packaging substrate; **(iv)** 3D images of three layers including SIBS tribo-layer, hydrogel layer, and SIBS substrate. Representative results are presented from $n = 3$ independently repeated experiments. **(b)** Transmittance measurement at UV and visible light regions for the soft film, hard film and whole device, showing their high optical transparency.

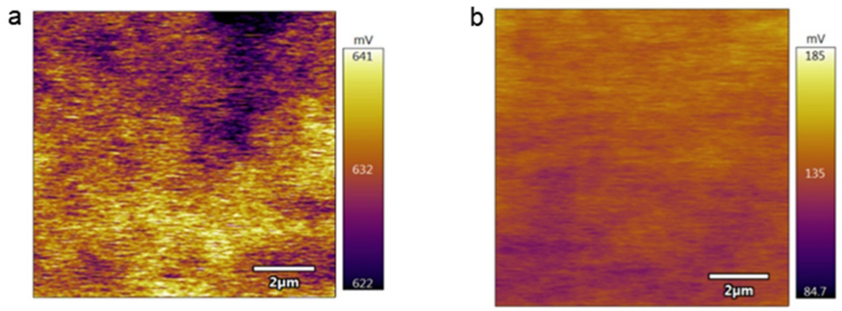

Fig. S14. Surface potential of **(a)** the SIBS film and **(b)** the PDMS film measured by KPFM. Higher surface potential was found on the SIBS film, suggesting that SIBS will lose electrons and PDMS will gain electrons when they contact with each other. Representative results are presented from n = 3 independently repeated experiments.

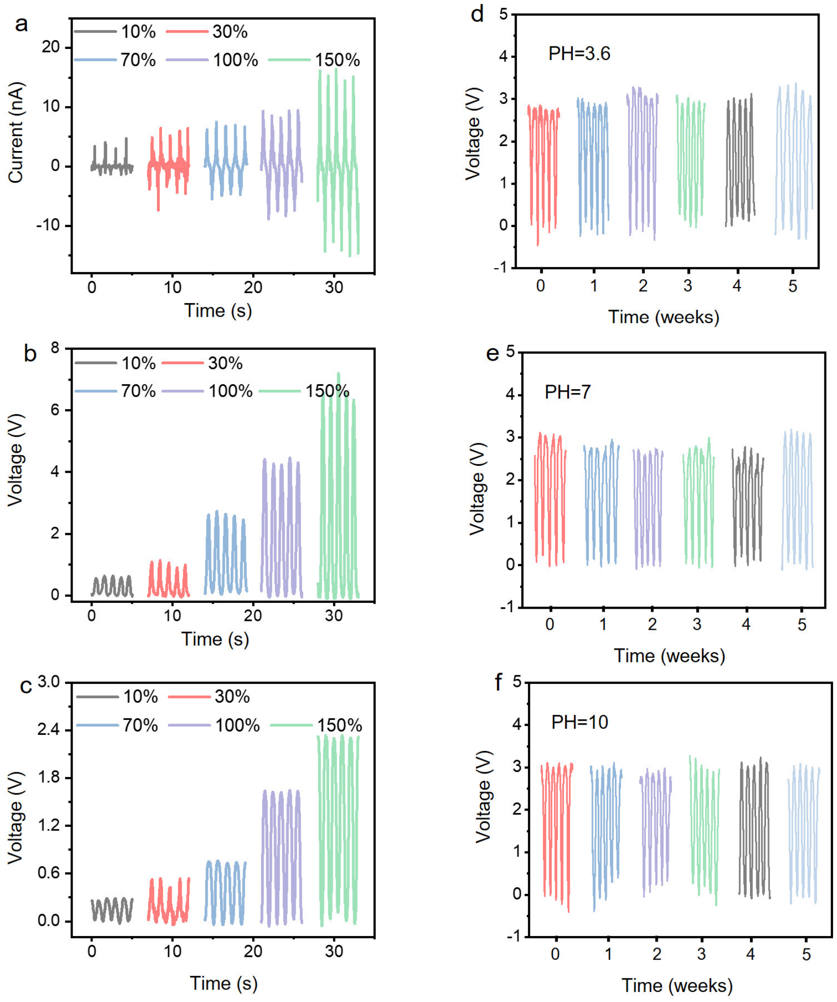

Fig. S15. Electrical outputs of the arbitrarily deformable device. **(a-c)** Current **(a)**, voltage **(b)**, and charge **(c)** variations as a function of the stretching degree. The device was stretched by a linear

motor at the frequency of 1 Hz. **(d-f)** Voltage outputs as a function of time when the device was fully immersed in PBS solutions with different PH values for 5 weeks, showing its high working stability in harsh chemical environments.

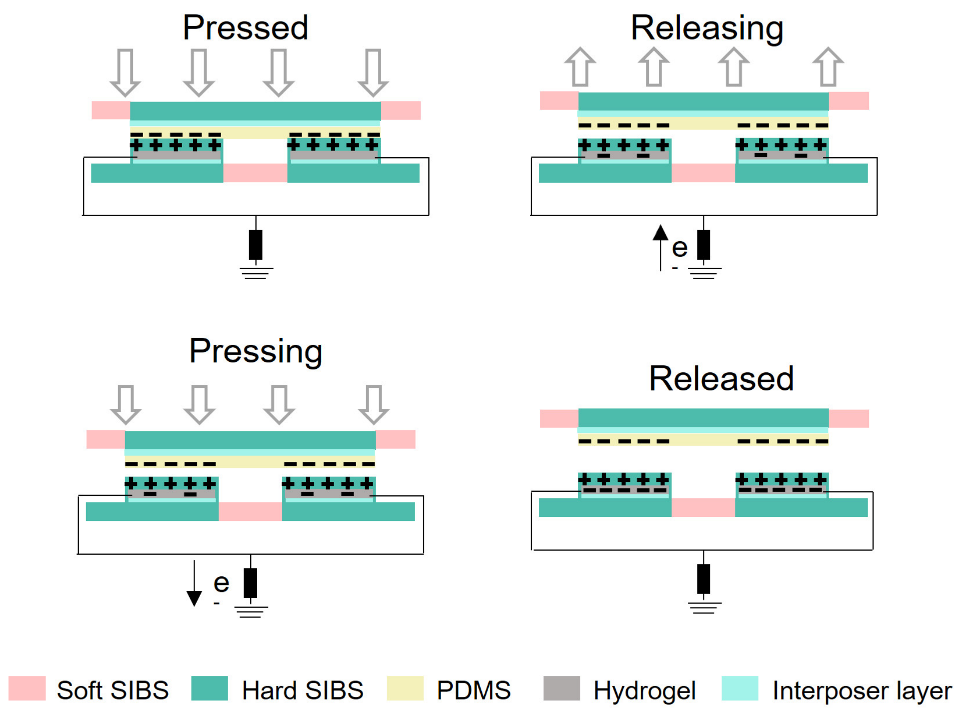

Fig. S16. Schematic of the working principle of the arbitrarily deformable device under pressing.

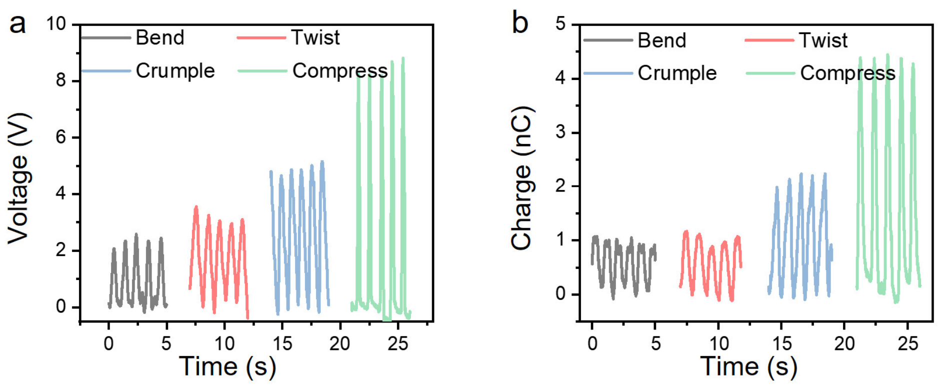

Fig. S17. Electrical outputs of the device generated by arbitrary deformations. **(a)** Voltage and **(b)** charge outputs generated by bending, twisting, crumpling and compressing.

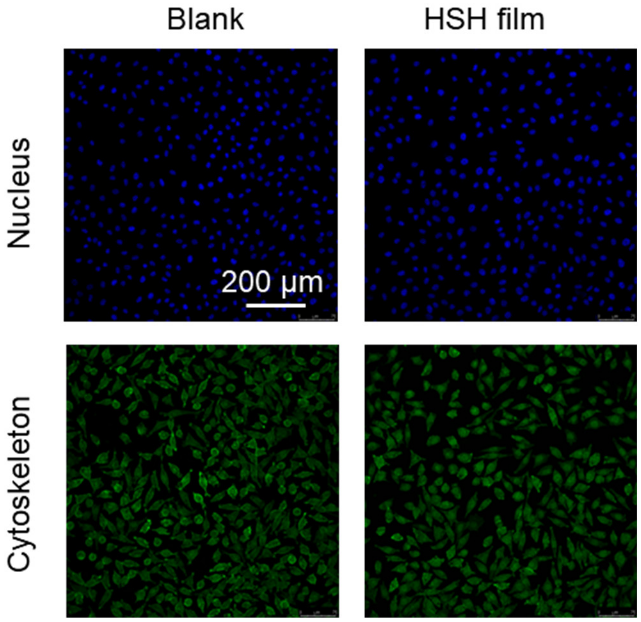

Fig. S18. Biocompatibility of the packaging film. Fluorescence micrographs for the morphology of L929 cells on a blank sample and a SHS packaging film (the well bottom of a 48-well plate). Blue indicates the nucleus stained with DAPI and green indicates the cytoskeleton stained with FICT Phalloidin. Representative results are presented from $n = 3$ independently repeated experiments.

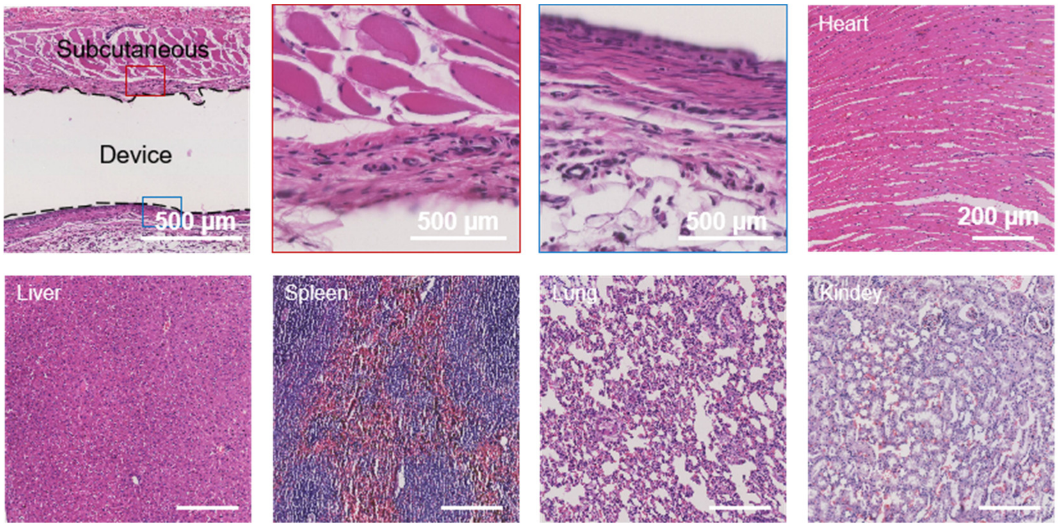

Fig. S19. Pathological analyses after 10-week implantation. The H&E-stained regional tissues surrounding the implanted device and on most vital organs, including heart, liver, spleen, lung, and kidney. No signs of inflammation were observed. Representative results are presented from $n = 3$ independently repeated experiments.

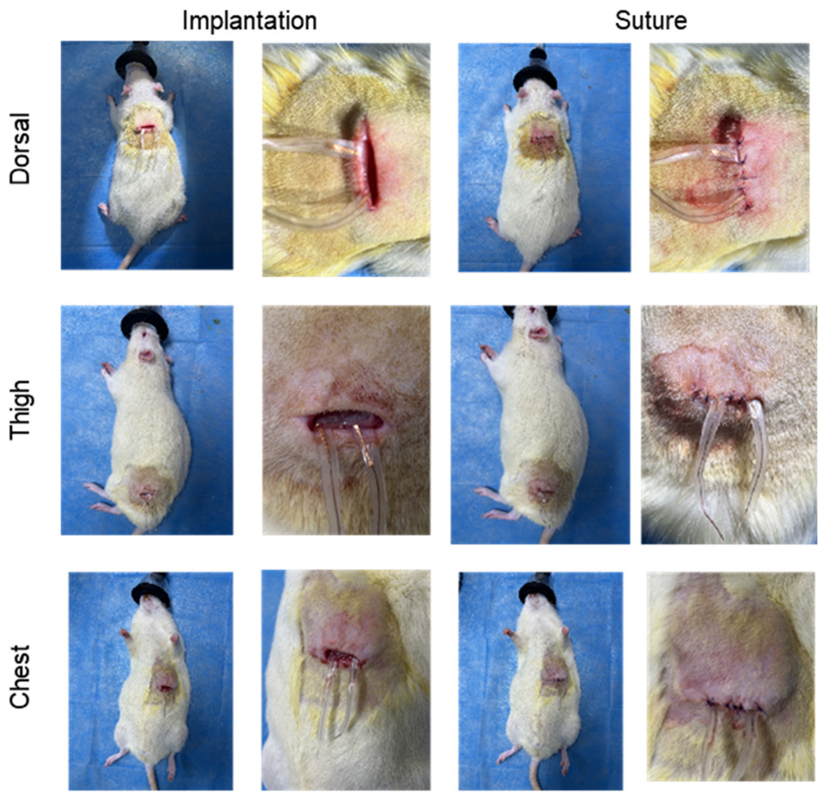

Fig. S20. Implantation process of the bioelectronic device in the dorsal, thigh and chest regions of SD rats. Skin incision was ~1.5 cm. The device was implanted between the epithelial and deep muscle layer. The incision was sutured after the implantation.

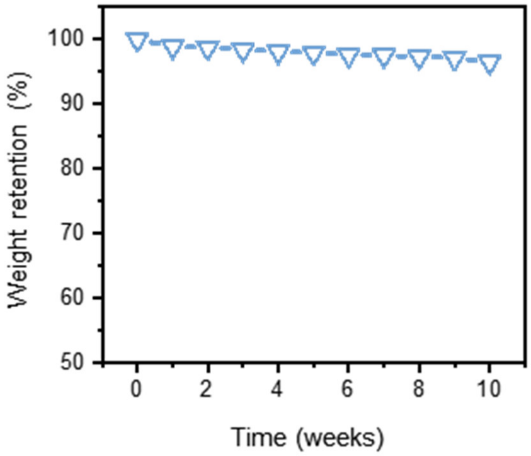

Fig. S21. The water retention property of SIBS packaged chitosan hydrogel at room temperature within 10 weeks. The weight fraction corresponds to the preserved/original weights.

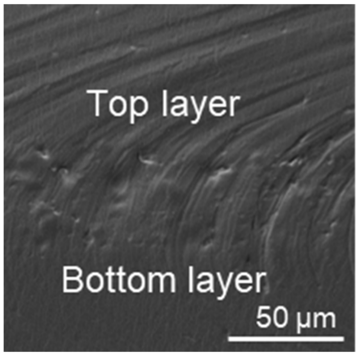

Fig. S22. Cross-section SEM image of the device edge, showing the seamless interface that prevents the edge leakage. Representative results are presented from $n = 3$ independently repeated experiments.

Supplementary Tables

Table S1. Comparison of packaging substrates with regional stretchability

Substrate	Strain (%)	Strategy	Modulus	Ref.
PDMS	300	Create rigid region by secondary cross-linking	0.17~0.6 MPa	1
PDMS	100	Create rigid region by UV exposure	0.12~2.76 MPa	2
PDMS	175	Create rigid region with transfering	0.12 MPa; 1.65 GPa	3
Ecoflex	70	Connect rigid and soft regions by welding	0.12~0.65 MPa	4
Ecoflex	150	Create rigid region by welding PDMS	50 kPa, 1.9 MPa	5
PU	10	Create rigid region by penetrating platelets	230 MPa; 1.3 GPa	6
PU	300	Create rigid region by penetrating platelets	4 MPa; 7GPa	7
PDMS	100	Create rigid region by embedding	0.2MPa;170GPa	8
SEBS	100	Connect rigid and soft regions by welding	1.5 MPa; 5 MPa	9
SEBS	500	Connect rigid and soft regions by welding	/	10
Polysulfone	24	Create rigid region by surface oxidating	5.3 MPa, 775 MPa	11
PDMS	70	Create rigid region with larger crosslink density	3 MPa; 160 MPa	12
PAAm-alginate	150	Create rigid region by locally stiffened hydrogel	40 kPa, 330 kPa	13
PDMS	40	Create rigid region by embedding	1 MPa; 1.5 GPa	14
PDMS	60	Create rigid region by embedding	1 MPa; 650 MPa	15
Ecoflex	100	Create rigid region by embedding	70 kPa, 2.7 GPa	16
PUA	60	Create rigid region by UV irradiation	/	17
PDMS	30	Create rigid region by partially perforated	0.5 MPa, 1GPa	18
Micro-holes	38	Introduce micro-holes	1 MPa	19
PDMS	20	Introduce protruding structure	1 MPa	20
PVDF-HFP	50	Create rigid region with microelectrode	93 kPa	21
SIBS	600	Create soft region with oligomer	60 kPa~3 MPa	This work

Abbreviations: polydimethylsiloxane (PDMS); Polyurethane (PU); Polystyrene–ethylene/butylene styrene)(SEBS); Polyacrylamide (PAAm); Polyurethane acrylate (PUA); Poly(vinylidene fluoride-co- hexafluoropropylene) (PVDF-HFP); Polystyrene-isobutylene-styrene (SIBS).

Table S2. Comparison of interfacial adhesions between module and stretchable substrate

Materials		Strain (%)	Strategy	Ref.
Stretchable substrate	Adhered film			
Ecoflex	Hydrogel	125	Chemical bonding	22
Ecoflex	Hydrogel	700	Chemical bonding	23
SEBS	SEBS	100	Topological entanglements	24
SEBS	SEBS	30	Topological entanglements	25
PDMS	Glass	\	Chemical bonding	26
Au/PDMS	Au/PDMS	35	Mechanical interlocking	27
SIS	Rigid unit	500	Mechanical interlocking	28
PDMS	PDMS	80	Chemical bonding	29
PDMS	PET	33	Chemical bonding	30
PDMS	Organogel/Hydrogel	250	Mechanical interlocking	31
SBS	SIS	100	Topological entanglements	32
SEBS	SEBS	100	Topological entanglements	33
SEBS	SEBS	20	Topological entanglements	34
PDMS	PDMS/Ag flakes	80	Topological entanglements	35
PDMS	PEDOT:PSS	100	Hydrogen bonding	36
PU	PEDOT:PSS	80	Molecular interdiffusion	37
PDMS	Butyl rubber	80	Introducing micro-holes	38
SBS	PDMS	100	Physical effect	39
Ecoflex	Ecoflex	50	Molecular interdiffusion	40
PDMS based film	PDMS/SEBS/PU	60	Covalently and non-covalently bonding	41
SIBS	Si/PET/PDMS/Al foil/ PEDOT: PSS/ hydrogel	600	Topological entanglements and chemical bonding	This work

Abbreviations: Polydimethylsiloxane (PDMS) ; Poly(styrene-isoprene-styrene) (SIS); Poly(styrene-butadiene-styrene) (SBS); Poly(styrene–ethylene/butylene–styrene) (SEBS); Polyurethane (PU); Poly(styrene-isobutylene-styrene) (SIBS);

Table S3. Comparison of flexible/stretchable implantable biosensors

Encapsulation materials	In-vivo durability	Deformation response	Function	Ref.
PDMS-parylene	48 h	Compress	Cardiac monitoring	42
PLA	5 days	Compress	Pressure sensor	43
PLGA	14 days	Compress	Stimulation of nerve cells	44
Kapton	48 h	Compress	Cardiac monitoring	45
PDMS	5 days	Compress	\	46
Polyimide/Epoxy	48 h	Compress	Motility sensing	47
PLA	2 weeks	Compress	Monitoring the nerve tissue repair	48
PDMS-parylene	30 days	Compress	Cardiac monitoring	49
PLA	4 days	Compres	Respiration monitoring	50
PDMS-parylene	72 h	Stretch	Biomedical monitoring	51
PDMS-parylene	4 weeks	Compres	Cardiac monitoring	52
PDMS	3 days	Compres	modifying fibroblast alignment	53
PDMS-parylene	5 days	Compress	Heart motion	54
PLA	3 weeks	Compress	Force sensor	55
PDMS	3 days	Compress	Heart motion	56
Silicon rubber	24 h	Compress	Cardiac pacemaker	57
Ecoflex	24 h	Stretch	Respiration monitoring	58
Parylene-C	3 days	Compress	Blood glucose control	59
Silicon	7 days	Stretching	Motion monitoring	60
PDMS	7 days	Compress	\	61
SIBS	10 weeks	Stretch/bend compress/twist	Respiration monitoring	This work

Abbreviations: polydimethylsiloxane (PDMS); Poly(lactic acid) (PLA) ; Poly(lactic-co-glycolic acid) (PLGA); Poly(styrene-isobutylene-styrene) (SIBS).

Supplementary References

1. Miao, L. et al. Localized modulus-controlled PDMS substrate for 2D and 3D stretchable electronics. *J. Micromech. Microeng.* **30**, 045001 (2020).

2. Cai, M., Nie, S., Du, Y., Wang, C., Song, J. Soft elastomers with programmable stiffness as strain-isolating substrates for stretchable electronics. *ACS Appl. Mater. Interfaces* **11**, 14340-14346 (2019).

3. Lee, Y. et al. Stretchable array of CdSe/ZnS quantum-dot light emitting diodes for visual display of bio-signals. *Chem. Eng. J.* **427**, 130858 (2022).

4.Yoon, J. et al. Design and fabrication of novel stretchable device arrays on a deformable polymer substrate with embedded liquid-metal interconnections. *Adv. Mater*. **26**, 6580-6586 (2014).

5. Moser, R. et al. From playroom to lab: tough stretchable electronics analyzed with a tabletop tensile tester made from toy-bricks. *Adv. Sci.* **3**, 1500386 (2016).

6. Erb, R. M. et al. Locally reinforced polymer-based composites for elastic electronics. *ACS Appl. Mater. Interfaces* **4**, 2860-2864 (2012)

7. Libanori, R. et al. Stretchable heterogeneous composites with extreme mechanical gradients. *Nat. Commun.* **3**, 1265 (2012).

8. Naserifar, N. et al. Material gradients in stretchable substrates toward integrated electronic functionality. *Adv. Mater.* **28**, 3584-3591 (2016).

9. Wang, W. et al. Strain-insensitive intrinsically stretchable transistors and circuits. *Nat. Electron.* **4**, 143-150 (2021).

10. Jiang, Y. et al. A universal interface for plug-and-play assembly of stretchable devices. *Nature* **614**, 456-462 (2023).

11. Cao, Y. et al. Direct fabrication of stretchable electronics on a polymer substrate with process-integrated programmable rigidity. *Adv. Funct. Mater.* **28**, 1804604 (2018).

12. Park, C. W. et al. Locally-tailored structure of an elastomeric substrate for stretchable circuits. *Semicond. Sci. Technol.* **31**, 025013 (2016)

13. Liu, H. et al. Spatially modulated stiffness on hydrogels for soft and stretchable integrated electronics. *Mater. Horiz.* **7**, 203-213 (2020)

14. Byun, J. et al. Fully printable, strain-engineered electronic wrap for customizable soft electronics. *Sci. Rep.* **7**, 45328 (2017).

15. Byun, J.; Chung, S.; Hong, Y. Artificial soft elastic media with periodic hard inclusions for tailoring strain-sensitive thin-film responses. *Adv. Mater.* **30**, 1802190 (2018).

16. Lim, Y. et al. Biaxially stretchable integrated array of high performance microsupercapacitors. *ACS Nano* **11**, 11639–11650 (2014)

17. Kim, Y.; Jun, S.; Ju, B. K.; Kim, J. W. Heterogeneous configuration of a Ag nanowire/polymer composite structure for selectively stretchable transparent electrodes. *ACS Appl. Mater. Interfaces* **9**, 7505-7514 (2017)
18. Lee, Y. K. et al. Chemical sensing systems that utilize soft electronics on thin elastomeric substrates with open cellular designs. *Adv. Funct. Mater.* **27**, 1605476 (2017)
19. Rong, Y. et al. Stretchability improvement of flexiable electronics by laser micro-drilling array holes in PDMS film. *Opt. Lasers Eng.***134**,160307 (2020)
20. Cantarella, G. et al. Design of engineered elastomeric substrate for stretchable active devices and sensors. *Adv. Funct. Mater.* **28**, 1705132 (2018)
21. Su Q. et al. A stretchable and strain-unperturbed pressure sensor for motion interference–free tactile monitoring on skins. *Sci. Adv.* **7**, eabi4563 (2021)
22. Liu, T. et al. Triboelectric-nanogenerator-based soft energy-harvesting skin enabled by toughly bonded elastomer/hydrogel hybrids. *ACS Nano* **12**, 2818-2826 (2018).
23. Yuk, H., Zhang, T., Parada, G.A., Liu, X., Zhao, X. Skin-inspired hydrogel-elastomer hybrids with robust interfaces and functional microstructures. *Nat. Commun.* **7**, 12028 (2016).
24. Wang W. et al. Strain-insensitive intrinsically stretchable transistors and circuits. *Nat. Electron.* **4**, 143-150 (2021).
25. Jiang Y. et al. A universal interface for plug-and-play assembly of stretchable devices. *Nature* **614**, 456-462 (2023).
26. Xiong L., Chen P., Zhou Q. Adhesion promotion between PDMS and glass by oxygen plasma pre-treatment. *J. Adhes. Sci. Technol.* **28**, 1046-1054 (2014).
27. Zhu, M.et al. A mechanically interlocking strategy based on conductive microbridges for stretchable electronics. *Adv. Mater.* **34**, e2101339 (2022).
28. Lopes, P.A., Santos, B.C., de Almeida, A.T., Tavakoli, M. Reversible polymer-gel transition for ultra-stretchable chip-integrated circuits through self-soldering and self-coating and self-healing. *Nat. Commun.* **12**, 4666 (2021).
29. Hwang, H. et al. Stretchable anisotropic conductive film (S-ACF) for electrical interfacing in high-resolution stretchable circuits. *Sci. Adv.* **7**, eabh0171 (2021).
30. Erlenbach, S. et al. Flexible-to-stretchable mechanical and electrical interconnects. *ACS Appl. Mater. Interfaces* **15**, 6005-6012 (2023).
31. Jing, T. et al. Interfacial roughness enhanced gel/elastomer interfacial bonding enables robust and stretchable triboelectric nanogenerator for reliable energy harvesting. *Small* **19**,2206528 (2023).
32. Song, W.J. et al. Stand-alone intrinsically stretchable electronic device platform powered by stretchable rechargeable battery. *Adv. Funct. Mater.* **30**, 2003608 (2020).

33. Wang, S. et al. Skin electronics from scalable fabrication of an intrinsically stretchable transistor array. *Nature* **555**, 83-88 (2018).
34. Molina-Lopez, F. et al. Inkjet-printed stretchable and low voltage synaptic transistor array. *Nat. Commun.* **10**, 2676 (2019).
35. Guo, W. et al. Matrix-independent highly conductive composites for electrodes and interconnects in stretchable electronics. *ACS Appl. Mater. Interfaces* **11**, 8567-8575 (2019).
36. Li, G. et al. PEDOT:PSS/grafted-PDMS electrodes for fully organic and intrinsically stretchable skin-like electronics. *ACS Appl. Mater. Interfaces* **11**, 10373-10379 (2019).
37. Lee, J. et al. Inter-diffused thermoplastic urethane-PEDOT:PSS bilayers with superior adhesion properties for high-performance and intrinsically-stretchable organic solar cells. *J. Mater. Chem. A* **11**, 12846-12855 (2023).
38. Vohra, A., Schlingman, K., Carmichael, R. S., Carmichael, T.B. Membrane-interface-elastomer structures for stretchable electronics. *Chem* **4**, 1673-1684 (2018).
39. You, I., Kong, M., Jeong, U. Block Copolymer Elastomers for Stretchable Electronics. *Acc. Chem. Res.* **52**, 63-72 (2019).
40. Jeong, K. et al. A sub-micron-thick stretchable adhesive layer for the lamination of arbitrary elastomeric substrates with enhanced adhesion stability. *Chem. Eng. J.* **429**,132250 (2022).
41. Kang, J. et al. Tough-interface-enabled stretchable electronics using non-stretchable polymer semiconductors and conductors. *Nat. Nanotechnol.***17**, 1265-1271 (2022).
42. Zheng, Q. et al. In vivo self-powered wireless cardiac monitoring via implantable triboelectric nanogenerator. *ACS Nano* **10**, 6510-6518 (2016).
43. Ouyang, H. et al. A bioresorbable dynamic pressure sensor for cardiovascular postoperative care. *Adv. Mater.* **33**, e2102302 (2021).
44. Zheng, Q. et al. Biodegradable triboelectric nanogenerator as a life-time designed implantable power source. *Sci. Adv.* **2**, e1501478 (2016).
45. Azimi, S. et al. Self-powered cardiac pacemaker by piezoelectric polymer nanogenerator implant. *Nano Energy* **83**,105781 (2021).
46. Yu, Y. et al. Biocompatibility and in vivo operation of implantable mesoporous PVDF-based nanogenerators. *Nano Energy* **27**, 275-281 (2016).
47. Dagdeviren, C, et al. Flexible piezoelectric devices for gastrointestinal motility sensing. *Nat. Biomed. Eng.* **1**, 807-817 (2017).
48. Wu, P. et al. Ultrasound-driven in vivo electrical stimulation based on biodegradable piezoelectric nanogenerators for enhancing and monitoring the nerve tissue repair. *Nano Energy* **102**, 107707 (2022).

49. Li J, et al. Long-term in vivo operation of implanted cardiac nanogenerators in swine. *Nano Energy* **90**, 106507 (2021).
50. Yang, F. et al. Wafer-scale heterostructured piezoelectric bio-organic thin films. *Science* **373**, 337–342 (2021).
51. Ma, Y. et al. Self-powered, one-stop, and multifunctional implantable triboelectric active sensor for real-time biomedical monitoring. *Nano Lett.* **16**, 6042-6051 (2016).
52. Xie, F. et al, An experimental study on a piezoelectric vibration energy harvester for self-powered cardiac pacemakers. *Ann. Transl. Med.* **9**, 880 (2021).
53. Wang, A. et al. Piezoelectric nanofibrous scaffolds as in vivo energy harvesters for modifying fibroblast alignment and proliferation in wound healing. *Nano Energy* **43**, 63-71 (2018)
54. Li, N. et al. Direct powering a real cardiac pacemaker by natural energy of a heartbeat. *ACS Nano* **13**, 2822-2830 (2019)
55. Cheng, Y. et al. Boosting the piezoelectric sensitivity of amino acid crystals by mechanical annealing for the engineering of fully degradable force sensors. *Adv. Sci.* **10**, e2207269 (2023)
56. Ouyang, H. et al. Symbiotic cardiac pacemaker. *Nat Commun.* **10**, 1821 (2019)
57. Ryu, H. et al. Self-rechargeable cardiac pacemaker system with triboelectric nanogenerators. *Nat Commun.* **12**, 4374 (2021)
58. Li, J. et al. implanted battery-free direct-current micro-power supply from in vivo breath energy harvesting. *ACS Appl. Mater. Interfaces* **10**, 42030-42038 (2018)
59. Liu, Z. et al. A self-powered optogenetic system for implantable blood glucose control. *Research* **2022**, 9864734 (2022)
60. Sheng, F. et al. Ultrastretchable organogel/silicone fiber-helical sensors for self-powered implantable ligament strain monitoring. *ACS Nano* **16**, 10958-10967 (2022)
61. Cheng, B. et al. Mechanically asymmetrical triboelectric nanogenerator for self-powered monitoring of in vivo microscale weak movement. *Adv. Energy Mater.* **10**, 2000827 (2020)
