# Supplementary material for: A universal packaging substrate for mechanically stable assembly of stretchable electronics
Source: Nat Commun. 2024 Jul 19;15:6106. doi: 10.1038/s41467-024-50494-8 (PMC11271615; doi:10.1038/s41467-024-50494-8)
Supplement: Supplementary file 3 — Description of Additional Supplementary Information [file 41467_2024_50494_MOESM3_ESM.docx]

**Description of Additional Supplementary Files**

File Name: Movie S1

Description: A largely stretchable operating circuit enabled by the packaging substrate

File Name: Movie S2

Description: Stretch-release cycling of the bioelectronic device.

File Name: Movie S3

Description: An implanted device in the chest to detect the respiration of a rat.

File Name: Movie S4

Description: An implanted device in the dorsal region to convert mechanical energy to electricity.

File Name: Movie S5

Description: An implanted device in the thigh to generate electricity from the movement of the thigh.
